# Scheimpflug Camera and Swept-Source Optical Coherence Tomography in Pachymetry Evaluation of Diabetic Patients

**DOI:** 10.1155/2019/4532657

**Published:** 2019-04-15

**Authors:** Katarzyna Krysik, Dariusz Dobrowolski, Karolina Stanienda-Sokół, Edward A. Wylegala, Anita Lyssek-Boron

**Affiliations:** ^1^Department of Ophthalmology with Pediatric Unit, St. Barbara Hospital, Trauma Center, Medykow Square 1, 41-200 Sosnowiec, Poland; ^2^Chair and Clinical Department of Ophthalmology, School of Medicine with the Division of Dentistry in Zabrze, Medical University of Silesia in Katowice, Panewnicka 65 St., 40-760 Katowice, Poland; ^3^Department of Ophthalmology, District Railway Hospital, Panewnicka 65 St., 40-760 Katowice, Poland; ^4^Hebei Provincial Eye Hospital, Xingtai, China

## Abstract

**Aim:**

The comparative analysis of the central and peripheral corneal thicknesses using two different imaging systems: Scheimpflug camera and swept-source OCT was performed to investigate the differences in corneal thickness analysis in diabetic patients.

**Materials and Methods:**

The study group consisted of the 147 eyes of 107 diabetic patients who were examined and compared with 138 eyes of 89 nondiabetic cataract patients. The inclusion criteria for the study group was diabetes mellitus type II identified no less than 10 years ago, with NPDR not requiring prior laser treatment. The control group was recruited from nondiabetic patients. Measurements were obtained on the Pentacam Scheimpflug imaging system and Casia swept-source OCT. All study parameters from anterior chamber images were processed for five different zones, the central zone and four peripherals—superior, inferior, nasal, and temporal. A fit zone diameter of 4 mm was applied for both instruments.

**Results:**

The Pentacam system overestimated corneal measurements in the DM group when compared with the Casia OCT in superior corneal zone (*p*=0.04), inferior corneal zone (*p*=0.02), nasal corneal zone (*p* < 0.001), and temporal corneal zone (*p*=0.01). In the control group, there were also statistically significant differences between the Pentacam and Casia OCT measured values in inferior corneal zone (*p*=0.001), nasal corneal zone (*p*=0.04), and temporal corneal zone (*p* < 0.001).

**Conclusion:**

Scheimpflug camera pachymetry measurements showed statistically higher CCT values when compared with swept-source OCT measurements.

## 1. Introduction

Diabetes mellitus (DM), one of the most common metabolic disorders worldwide, is associated with many ocular complications. First of all, diabetic retinopathy (DR) affects the retinal vessels and is divided into two main groups, namely, nonproliferative diabetic retinopathy (NPDR) and proliferative diabetic retinopathy (PDR) [1, 2]. Patients with DM are also predisposed to damage of all layers of the cornea. Morphological changes of the cornea include the corneal endothelium playing a vital role in keeping the stroma dehydrated [3]. The damage manifests in decreased endothelial cell density, polymorphism, and polymegathism [4–6]. Also, the reduced density of basal epithelial cells, associated with haemoglobin A1c and advanced glycation end products, plays important role in disorders of the corneal surface [7, 8]. The previously mentioned disorders lead to endothelial dysfunction and differences in corneal thickness [4, 5, 7–9]. It may lead to changes in the refractive errors and corneal transparency. DM is responsible for damage of the pericytes and vascular endothelium causing reduced blood supply to Schwann cells or neurons [3, 10]. Neurotrophic loss of corneal sensation leads to reduced tear production and its consequences like dryness of the eye, punctate keratitis, persistent epithelial defects, or impaired corneal sensitivity, so-called diabetic keratopathy [3, 10, 11]. Structural and functional changes in corneas contribute to increased surgical risk, complications, and prolonged corneal healing [1, 3, 5, 10].

Other ophthalmic manifestations of DM include changes in lens transparency and premature cataract development, altered pharmacological mydriasis, orbital and lid features like cranial nerve palsies, chalazia, xanthelasma, and cellulitis, and conjunctival abnormalities comprising pterygia, pinguecula, tortuosity, and dilation of conjunctival vessels [11, 12].

The imaging of anterior chamber structures should be estimated with objective qualitative and quantitative methods. Corneal thickness can be measured using different devices, such as Scheimpflug camera imaging, optical pachymetry, confocal microscopy, ultrasound biomicroscopy, scanning slit topography, scanning peripheral anterior chamber depth analyser, time-domain optical coherence tomography (OCT), or ultrasound pachymetry [13–16]. Until recently, ultrasound was considered the gold standard in pachymetry, and most ophthalmologists are familiar with this device. However, despite common accessibility, this method has limitations. It is not a global pachymetry measurement, as only specific points can be measured, and it requires local anaesthesia and aseptic precautions [13, 14].

The aim of this study was the comparative analysis of the central and peripheral corneal thicknesses using two different imaging systems of measurement, the Pentacam Scheimpflug camera and Casia swept-source OCT, to investigate the effect of DM type II with DR not requiring photocoagulation on corneal thickness.

## 2. Materials and Methods

The study was performed at the Ophthalmology Department of Saint Barbara Hospital, Trauma Centre, Sosnowiec, Poland. The study was conducted under tenets of the Declaration of Helsinki. All patients signed an informed consent form before ophthalmic examination and surgical procedures.

### 2.1. Participants

Patients from the study and control groups were recruited from cataract patients operated on between January 1, 2018, and September 30, 2018. Basic information was collected from all patients including age, sex, medical history, and duration of diabetes mellitus. Complete ophthalmic examinations including preoperative best-corrected Snellen distance visual acuity, intraocular pressure (IOP) measurement using Goldmann applanation tonometry, slit-lamp biomicroscopy, and fundus examination with a dilated pupil were performed. Randomisation of groups was done according to inclusion/exclusion criteria. The inclusion criteria for the study group was DM type II identified no less than 10 years ago, with NPDR not requiring prior laser photocoagulation. The control group was recruited from nondiabetic cataract patients. The exclusion criteria for both patient groups were all systemic and ophthalmic conditions likely to affect the corneal state and thickness. This included corneal pathologies such as scars and haze, degenerations and dystrophies, pseudoexfoliation syndrome, previous ocular surgeries (mainly corneal refractive surgery) or ocular trauma, ocular hypertension or glaucoma, uveitis, contact lens wearers, cornea-depending refractive error (±4.0 spherical dioptres and ±2.5 cylindrical dioptres), usage of topical medication which may affect the ocular surface and corneal condition (mainly medications with preservatives), and systemic diseases with ocular involvement, like autoimmune or inflammatory diseases. Randomisation was done during routine admissions to the hospital.

The study group consisted of the 147 eyes of 107 diabetic patients who were examined and compared with 138 eyes of nondiabetic 89 cataract patients. All measurements submitted for the study were obtained prior to cataract surgery.

Measurement of corneal thickness was determined by one operator using two different imaging systems, the Pentacam Scheimpflug imaging system and Casia swept-source OCT. All study parameters from anterior chamber images were processed for five different zones, the central zone and four peripherals—superior, inferior, nasal, and temporal. A fit zone diameter of 4 mm was applied for both instruments. Two consecutive measurements were obtained for each eye of each patient for each device.

### 2.2. Instruments

The Pentacam Scheimpflug imaging system (Pentacam HR, Oculus, Wetzlar, Germany) uses rotating cameras to reconstruct the three-dimensional structure of the cornea from two-dimensional optical sections, which provide sharp images for detailed analysis from the anterior corneal surface through the posterior aspect of the crystalline lens. It uses a 475 nm wavelength blue light-emitting diode (LED) to provide anterior and posterior surface topography of the cornea, pachymetry, anterior chamber angle, depth, and volume data as well as crystalline lens analysis (densitometry). The instrument-based software allows automatic analysis of various anterior segment parameters and takes 25 images per measurement within two seconds. It captures 100 slit images with a slip depth of 14.0 mm in 2 s by rotating along the optical axis from 0° to 360°. Central corneal thickness (CCT) is measured as the difference between anterior and posterior elevations in the central cornea [13, 15, 16].

Swept-source OCT (Casia SS-1000, Tomey, Nagoya, Japan) is a swept-source anterior segment OCT that uses a wavelength of 1310 nm and performs measurements with a speed of 30,000 axial scans per second. In the corneal map mode, each 3D image consists of 16 B-scans and 512 A-lines, and in the anterior segment mode, each 3D scan contains 128 B-scans and 512 A-scans. Total scan duration is 0.3 s for measurement of corneal thickness and corneal topography. The software automatically analyses the recorded images and provides various corneal maps, as well as a quantitative and qualitative anterior segment structure evaluation [17–19].

The axial resolution, offered by both noncontact devices, is 10 *μ*m for Pentacam-Scheimpflug camera and 10 *μ*m for CASIA OCT .

### 2.3. Statistical Analysis

The computer software XLSTAT-Biomed (Addinsoft SARL, France) was used for statistical analysis and to calculate means and standard deviation. The parameter values were compared between the control and DM groups using the Student's *t*-test or Mann–Whitney *U* test. In a Bland–Altman plot, the difference between measurements with different methods is plotted against their mean. The 95% limit of agreement (mean difference ± 1.96 standard deviation) provides the distance between measurements with 95% confidence. The Bland–Altman plot also shows proportional bias in the measurements, which is the relationship of the difference between measurements and the true value. A value less than 0.05 was considered statistically significant.

## 3. Results

Between January 1, 2018, and September 30, 2018, 107 diabetic patients (55 females and 52 males), with NPDR not requiring prior laser photocoagulation, and 89 nondiabetic patients (46 females and 43 males) underwent phacoemulsification surgery with in-the-bag intraocular lens implantation. The mean age of the study group was 71.85 ± 8.04 years (range 49–88 years old) and of the control group was 69.08 ± 9.13 years (range 45–84 years old). There was no statistically significant difference with respect to gender or age between the groups. In the DM group, the disease was recognized during routine glucose level tests, performed by GPs usually in every year.

Table 1 shows average pachymetry and the standard deviation of five different corneal zones measured by two different systems, the Pentacam Scheimpflug imaging system and CASIA swept-source OCT, in diabetic and control group patients, respectively.

The Pentacam overestimated corneal measurements in the DM group when compared with the Casia: superior corneal zone (*p*=0.04), inferior corneal zone (*p*=0.02), nasal corneal zone (*p* < 0.001), and temporal corneal zone (*p*=0.01). In the control group, there were also statistically significant differences between the Pentacam and Casia measured values: inferior corneal zone (*p*=0.001), nasal corneal zone (*p*=0.04), and temporal corneal zone (*p* < 0.001).

Central and nasal corneal zone thicknesses measured with both methods had a statistically significant difference. On the contrary, measurements of the temporal corneal zone with both scanning methods had no statistically significant values. Statistically significant differences were also observed between Pentacam measurements for the inferior corneal zone and between Casia measurements for the superior corneal zone.

**Figure 1:**
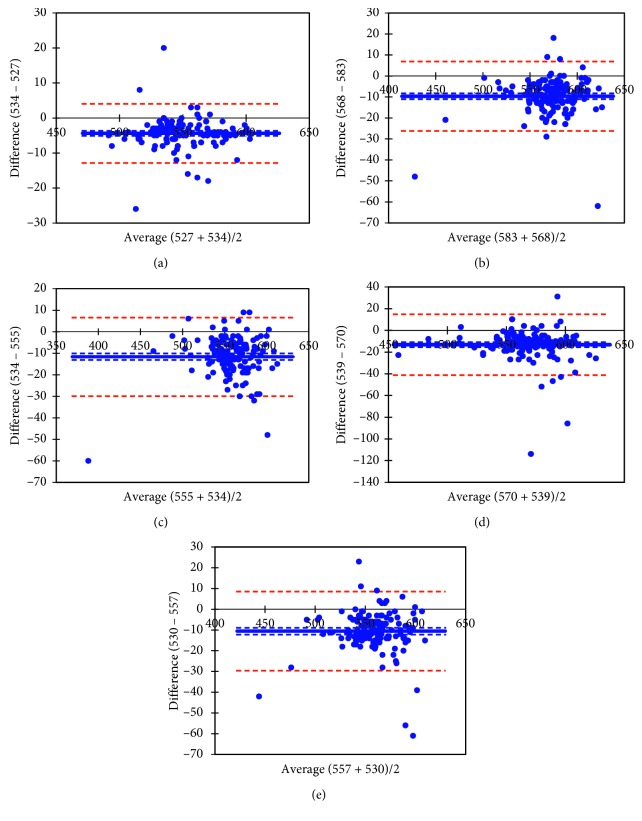
Bland–Altman plots showing agreement between Pentacam and Casia pachymetry measurements in five different corneal zones for the study group. (a) Central corneal thickness (bias = −4.37 ± 4.29); (b) superior corneal zone (bias = −9.66 ± 8.49); (c) inferior corneal zone (bias = −11.61 ± 9.29); (d) temporal corneal zone (bias = −13.18 ± 14.3); (e) nasal corneal zone (bias = −10.52 ± 9.73).

The Bland–Altman plot illustrates the level of agreement between the two instruments for each scan type, as well as the mean of the difference between evaluations generated by the two instruments, Pentacam and Casia, in the study group (Figure 1).

## 4. Discussion

The term “diabetic eye” is mostly thought to refer to the retinal, not corneal, pathology. Different corneal imaging systems are used to identify corneal pathologies and their progression. An accurate corneal thickness evaluation is crucial for IOP measurement prior to corneal and many other types of ocular surgery [9, 14, 20–23].

In our study, we compared two noncontact corneal thickness measurement devices, the Pentacam Scheimpflug imaging system and CASIA swept-source OCT, in diabetic patients with NPDR not requiring prior laser photocoagulation and with nondiabetic cataract patients.

Different studies postulate the influence of hyperglycaemia on endothelial dysfunction with consistent stromal hydration and swelling of the cornea [2, 5, 8, 24].

The results obtained by Elflein et al. [25], like Kotecha et al. [26], show no association between CCT and diabetes. Nevertheless, that the average CCT is significantly higher in diabetic versus nondiabetic patients is commonly underlined by different researchers. However, the interpretation of those results should include the type and duration of DM, type of retinal changes, and the method of treatment [6, 22, 24, 27–29]. The results of Senćanić et al. [29] show no significant difference in CCT between diabetic patients without diabetic retinopathy and NPDR, but a statistically significant difference in CCT between patients without diabetic retinopathy and PDR. The highest mean CCT values in this study were recorded in the PDR patients, followed by the NPDR group.

Qu et al. [7] divided the cornea into five zones (central, superior, inferior, nasal, and temporal), as in the current study. They evaluated parameters affecting corneal thickness—endothelial cells, basement epithelial cells, and sub-basal nerve plexus. The central endothelial cell density was not significantly different in the diabetic patients and healthy controls. This is contrary to many other findings [5, 6, 24, 30].

Hashemi et al. [31] compared central and peripheral corneal thicknesses between diabetic and nondiabetic patients during a five-year period using the Pentacam. The diabetic group showed less reduction in all corneal thickness zones than the nondiabetic group.

Sanchis-Gimeno et al. [21] evaluated differences in central and four midperipheral corneal thicknesses between type II diabetic patients and nondiabetic patients using the Orbscan Topography System II. Our results for the study group, both from the Pentacam and Casia, are compatible with reference to those authors' findings. Our control group results are partly different only in the Pentacam measurement group. The superior and nasal corneal thicknesses are comparable, and the temporal corneal zone has a greater value than the inferior.

But, an exact comparison of corneal central and peripheral pachymetry values using different measuring methods in diabetic individuals was not possible due to a lack of data in the reviewed literature.

The differences in values of measured parameters are also dependent on measuring methods and devices. Ultrasonic pachymetry, considered the gold standard for pachymetry, carries the risk of development of corneal epithelial defects and transmission of infection. Contemporary noncontact systems offer repeatability and a range of quantitative and qualitative information. Pentacam Scheimpflug differs in many aspects when compared with Casia. However, scan quality and axial resolution do not make this device worse in corneal thickness assessment.

In our study, Pentacam pachymetry measurements indicated statistically higher CCT values when compared with Casia measurements. These measurements are comparable with results reported in other studies [13, 18, 32]. Otherwise, the results of the Choo et al.'s [4] study revealed that however endothelial cell density is reduced and polymorphism and polymegathism are increased, CCT is unaffected.

There are several limitations of our study. Diabetes mellitus is not a homogenous disease, it has different stages, ocular and systemic complications and associations; therefore, the need for further studies cannot be overemphasised. Each factor should be taken into account when measuring corneal anatomical and biomechanical parameters.

In conclusion, the results of our study show the CCT in patients diagnosed no less than 10 years ago, with NPDR not requiring prior laser photocoagulation, is significantly higher than that in healthy individuals. This finding should be taken into account when measuring the IOP, diagnosing intraocular hypertension or glaucoma, or before any ocular surgery. Routine CCT measurement in diabetic patients may also be beneficial in the evaluation and treatment of diabetic keratopathy as well as corneal neuropathy.

## Figures and Tables

**Table 1:** Mean pachymetry values by corneal regions for the study groups.

Value (*μ*m) and region						
Technique	Group	Central	Superior	Inferior	Nasal	Temporal
Pentacam	DM	552 ± 23	577 ± 26	564 ± 27	575 ± 26	563 ± 25
	Control	543 ± 26	578 ± 20	559 ± 22	578 ± 21	561 ± 22
	*p* value	0.005	0.865	0.018	0.01	0.2

Casia OCT	DM	548 ± 22	567 ± 27	552 ± 28	562 ± 25	553 ± 25
	Control	541 ± 29	573 ± 23	551 ± 22	572 ± 20	549 ± 20
	*p* value	0.041	0.031	0.431	<0.001	0.119

## Data Availability

The data used to support the findings of this study are available from the corresponding author upon request.
